# A Clinical Note

**Published:** 1888-06

**Authors:** 


### A CLINICAL NOTE.

ARUM TRI., picks at lips or nose, one spot, till it bleeds; or at finger tips. Mouth excessively sore and raw.

CONIUM, constantly picks his nose, which bleeds easily, or picks his fingers; lies in bed most of the time; does not like to answer questions.

HELLEBORE, constantly picking his lips and clothes.

LACHESIS, picks fingers raw, or face, or arm; or picks threads from shawl or bed covers.

SELENIUM, picks his teeth until they bleed; inclined to bore fingers in nose.

STRAMONIUM, automatic grasping of hands to head, nose, ears, etc.

The following case, reported by the late R. R. Gregg, M. D., is very instructive:

I was called to a boy of six years of age, who had been suffering ten days from inflammation of the brain, evidently without effusion, and had been under the care, from the first, of a homœopathic physician, who had been giving low potencies and doses at short intervals. This patient, too, was entirely unconscious and had been several days; and in addition had had severe convulsions, which increased in frequency and severity until a day or two after my being called, when he had successive convulsions for one entire day, and so severe that once, when his attendants left him for a minute or two, he was so suddenly and so violently convulsed that he was thrown clear of the bed upon the floor. Constipation had existed from the first, and he had one peculiar symptom that may be of interest to mention. This was a continual boring with the left forefinger under the right *ala nasi* until he bored a hole a third to half an inch in diameter entirely through the lip at that point, on the teeth and gum. All efforts to hold or bind his hand would at once bring on a convulsion, so that we had to desist from that, and allow him to go on with this work.

The treatment was entirely with the high potencies—the 2000th and upward—and doses at not less than twelve hours interval on two or three occasions, while all the rest of the time they were given at twenty-four to forty-eight, or more, hours intervals. The remedies administered were *Nux vomica*, *Hyoscyamus*, *Cuprum*, *Helleborus*, *Belladonna*, etc., but none of these appeared to have the least effect until *Belladonna* was prescribed, after the first dose of which, in the 2000th potency, the patient improved steadily and markedly for two or three days, when a second dose was given, which entirely completed the cure of the case, and I discharged the patient well, excepting some remaining debility, in ten days to a fortnight from my first call. Nor was there any impairment of mind, defects of vision, or other annoying sequelæ of the case, but a complete restoration to health, which is a no less remarkable and happy result of such treatment than the safe relief at the time of such violent symptoms.

